# Bis(μ-ferrocene­carboxyl­ato)bis­[aqua­­bis(ferrocene­carboxyl­ato)methano­l­erbium(III)] methanol disolvate

**DOI:** 10.1107/S1600536811051245

**Published:** 2011-12-03

**Authors:** Jianmin Liu, Yuanyuan Li, Dacheng Li

**Affiliations:** a School of Chemistry and Chemical Engineering, Liaocheng University, Shandong 252059, People’s Republic of China

## Abstract

In the centrosymmetric title coordination compound, [Er_2_{Fe(C_5_H_5_)(C_6_H_4_O_2_)}_6_(CH_3_OH)_2_(H_2_O)_2_]·2CH_3_OH, the two Er^III^ ions are bridged by two ferrocene­carboxyl­ate anions as asymmetrically bridging ligands, leading to dimeric cores. The Er^III^ ion has a distorted dodeca­hedral coordination with six coordinating O atoms derived from the ferrocene­carboxyl­ate ligands and two coordinated O atoms from one water mol­ecule and one methanol mol­ecule. The asymmetric unit comprises a half of the complex mol­ecule and a methanol solvent mol­ecule. Intra­molecular O—H⋯O and C—H⋯O inter­actions occur. In the crystal, mol­ecules are linked by inter­molecular O—H⋯O hydrogen bonds and C—H⋯O as well as C—H⋯π inter­actions.

## Related literature

For related structures, see: Hou *et al.* (2003 ▶); Li *et al.* (2004 ▶). 
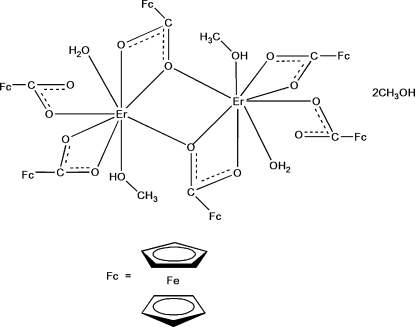


## Experimental

### 

#### Crystal data

[Er_2_Fe_6_(C_5_H_5_)_6_(C_6_H_4_O_2_)_6_(CH_4_O)_2_(H_2_O)_2_]·2CH_4_O
*M* *_r_* = 1872.91
Triclinic, 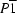

*a* = 12.0562 (14) Å
*b* = 12.115 (3) Å
*c* = 13.3198 (17) Å
α = 80.773 (2)°
β = 74.889 (1)°
γ = 66.153 (1)°
*V* = 1714.6 (5) Å^3^
*Z* = 1
Mo *K*α radiation
μ = 3.72 mm^−1^
*T* = 298 K
0.21 × 0.19 × 0.18 mm

#### Data collection

Bruker SMART CCD area-detector diffractometer
Absorption correction: multi-scan (*SADABS*; Sheldrick, 1996 ▶) *T*_min_ = 0.509, *T*_max_ = 0.554
9011 measured reflections
5965 independent reflections
4840 reflections with *I* > 2σ(*I*)
*R*_int_ = 0.023

#### Refinement

*R*[*F* ^2^ > 2σ(*F* ^2^)] = 0.033
*wR*(*F* ^2^) = 0.081
*S* = 1.00
5965 reflections
435 parameters
12 restraints
H-atom parameters constrained
Δρ_max_ = 0.98 e Å^−3^
Δρ_min_ = −0.64 e Å^−3^

### 

Data collection: *SMART* (Bruker, 2007 ▶); cell refinement: *SAINT* (Bruker, 2007 ▶); data reduction: *SAINT*; program(s) used to solve structure: *SHELXS97* (Sheldrick, 2008 ▶); program(s) used to refine structure: *SHELXL97* (Sheldrick, 2008 ▶); molecular graphics: *SHELXTL* (Sheldrick, 2008 ▶); software used to prepare material for publication: *SHELXTL*.

## Supplementary Material

Crystal structure: contains datablock(s) I, global. DOI: 10.1107/S1600536811051245/kp2371sup1.cif

Structure factors: contains datablock(s) I. DOI: 10.1107/S1600536811051245/kp2371Isup2.hkl

Additional supplementary materials:  crystallographic information; 3D view; checkCIF report

## Figures and Tables

**Table 1:** Selected bond lengths (Å)

Er1—O5	2.247 (3)
Er1—O1	2.280 (3)
Er1—O8	2.311 (3)
Er1—O2^i^	2.323 (3)
Er1—O7	2.329 (4)
Er1—O3	2.368 (4)
Er1—O4	2.381 (4)
Er1—O1^i^	2.667 (3)

Symmetry code: (i) 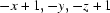.

**Table 2:** Hydrogen-bond geometry (Å, °) *Cg*1 is the centroid of the [please define] ring.

*D*—H⋯*A*	*D*—H	H⋯*A*	*D*⋯*A*	*D*—H⋯*A*
C20—H20⋯*Cg*1^ii^	0.93	3.18	3.823 (8)	128
O7—H7*C*⋯O4^i^	0.85	1.93	2.765 (5)	168
O8—H8*A*⋯O6	0.82	1.79	2.574 (5)	160
O9—H9⋯O6^iii^	0.82	1.87	2.682 (6)	172
C7—H7⋯O4^i^	0.93	2.55	3.465 (7)	167
C29—H29⋯O3	0.93	2.60	3.414 (7)	147
C34—H34*A*⋯O2^i^	0.96	2.54	3.051 (9)	114

Symmetry codes: (i) 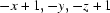; (ii) 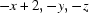; (iii) 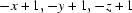.

**Fig. 1.**
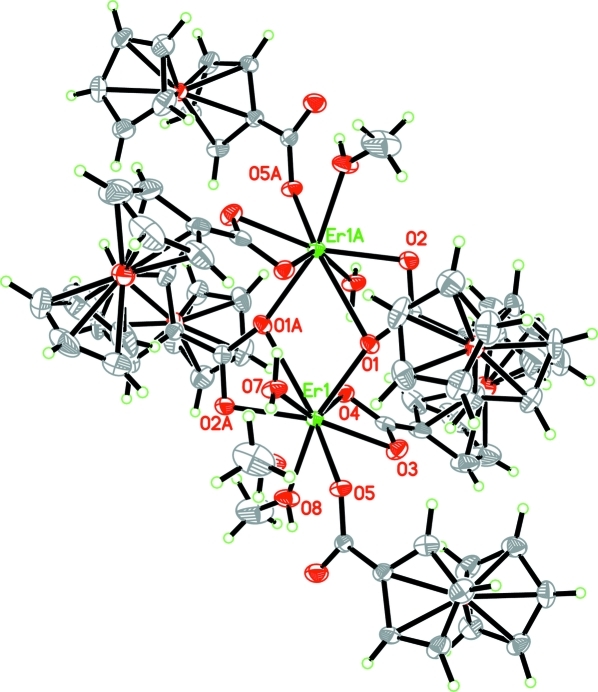
The asymmetric unit of the title compound, showing 30% probability displacement ellipsoids. Symmetry code for atoms: -x + 1, -y, -z + 1.

**Fig. 2.**
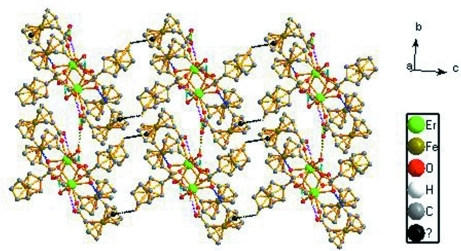
View of the one-dimensional chain structure in the title compound. H atoms have been omitted for clarity. Symmetry code for atoms: (i) -x+1, -y, -z+1; (ii) -x+2, -y, -z; (iii) -x+1, -y+1, -z+1.

## supplementary crystallographic information

### Comment

In the title compound (Fig. 1, Table 1), the averge bond distance of Er—O is 2.363Å which is shorter than Gd—O, Nd—O and Y—O in the simlar complexes (Hou *et al.*, 2003) and is longer than La—O in the simlar complex (Li *et al.*, 2004). The Er atom is eight-coordinated in a slightly distorted dodecahedron coordination geometry, with six coordinated oxygen atoms provided by ferrocenecarboxylate ligands and two coordinated oxygen atoms from one coordinated water and one coordinated methanol. Furthermore, six ferrocenecarboxylate ligands can be described as three groups (I, II and III). Group I contains two ferrocenecarboxylate ligands acting in tridentate fashion. O1 (or O1A) bridges two metal atoms (Er1 and Er1A), and O2 (or O2A) only coordinates one metal atom Er1 (or Er1A). The two ferrocenecarboxylate ligands belong to group II, in which two oxygen atoms from each ferrocenecarboxylate ligand chelate the Er1 (or Er1A) atom forming four-membered rings. Group III contains two ferrocenecarboxylate ligands acting in unidentate coordination mode. O5 (or O5A) only coordinates to one metal atom Er1 (or Er1A). The crystal packing is through C—H···O intramolecular hydrogen bond interactions. (Table 2, Fig.2).

### Experimental

Ferrocenecarboxylic Acid (0.3 mmol) and tetramethyl ammonium hydroxide (0.3 mmol) were added to a stirred solution of methanol (10 ml) in a Schlenk flask and stirred for 10 min. Then erbium nitrate pentahydrate (0.2 mmol) and sodium dicyandiamide (0.3 mmol) were added to the reactor and the reaction mixture was stirred for 10 h. The resulting clear solution was evaporated at room temperature. The product crystallizes as yellow crystals after one month. The title compound (yield 60%, m.p. 593–598 K). Anal. Calcd (%) for C_70_H_74_Er_2_Fe_6_O_18_(Mr = 1872.91): C, 44.89; H, 3.98. Found (%): C, 44.86; H, 4.00.

### Refinement

The H atoms were positioned geometrically, with methyl C—H distances of 0.96 Å, aromatic C—H distances of 0.93 Å, and refined as riding on their parent atoms, with *U*_iso_(H) = 1.5*U*_eq_(C) for the methyl groups, *U*_iso_(H) = 1.2*U*_eq_(C) for the cyclopentadienyl groups. While those of the water molecule were located from the Fourier map, constraining the O—H distances at 0.85 Å.

### Crystal data

**Table:**

[Er_2_Fe_6_(C_5_H_5_)_6_(C_6_H_4_O_2_)_6_(CH_4_O)_2_(H_2_O)_2_]·2CH_4_O	*Z* = 1
*M**_r_* = 1872.91	*F*(000) = 930
Triclinic, *P*1	*D*_x_ = 1.814 Mg m^−^^3^
Hall symbol: -P 1	Mo *K*α radiation, λ = 0.71073 Å
*a* = 12.0562 (14) Å	Cell parameters from 3903 reflections
*b* = 12.115 (3) Å	θ = 2.2–26.0°
*c* = 13.3198 (17) Å	µ = 3.72 mm^−^^1^
α = 80.773 (2)°	*T* = 298 K
β = 74.889 (1)°	Block, yellow
γ = 66.153 (1)°	0.21 × 0.19 × 0.18 mm
*V* = 1714.6 (5) Å^3^	

### Data collection

**Table:**

Bruker SMART CCD area-detector diffractometer	5965 independent reflections
Radiation source: fine-focus sealed tube	4840 reflections with *I* > 2σ(*I*)
graphite	*R*_int_ = 0.023
phi and ω scans	θ_max_ = 25.0°, θ_min_ = 1.6°
Absorption correction: multi-scan (*SADABS*; Sheldrick, 1996)	*h* = −14→11
*T*_min_ = 0.509, *T*_max_ = 0.554	*k* = −14→14
9011 measured reflections	*l* = −15→15

### Refinement

**Table:**

Refinement on *F*^2^	Primary atom site location: structure-invariant direct methods
Least-squares matrix: full	Secondary atom site location: difference Fourier map
*R*[*F*^2^ > 2σ(*F*^2^)] = 0.033	Hydrogen site location: inferred from neighbouring sites
*wR*(*F*^2^) = 0.081	H-atom parameters constrained
*S* = 1.00	*w* = 1/[σ^2^(*F*_o_^2^) + (0.0386*P*)^2^ + 1.2739*P*] where *P* = (*F*_o_^2^ + 2*F*_c_^2^)/3
5965 reflections	(Δ/σ)_max_ = 0.001
435 parameters	Δρ_max_ = 0.98 e Å^−^^3^
12 restraints	Δρ_min_ = −0.64 e Å^−^^3^

### Special details

**Table:**

Geometry. All e.s.d.'s (except the e.s.d. in the dihedral angle between two l.s. planes) are estimated using the full covariance matrix. The cell e.s.d.'s are taken into account individually in the estimation of e.s.d.'s in distances, angles and torsion angles; correlations between e.s.d.'s in cell parameters are only used when they are defined by crystal symmetry. An approximate (isotropic) treatment of cell e.s.d.'s is used for estimating e.s.d.'s involving l.s. planes.
Refinement. Refinement of *F*^2^ against ALL reflections. The weighted *R*-factor *wR* and goodness of fit *S* are based on *F*^2^, conventional *R*-factors *R* are based on *F*, with *F* set to zero for negative *F*^2^. The threshold expression of *F*^2^ > σ(*F*^2^) is used only for calculating *R*-factors(gt) *etc*. and is not relevant to the choice of reflections for refinement. *R*-factors based on *F*^2^ are statistically about twice as large as those based on *F*, and *R*- factors based on ALL data will be even larger.

### Fractional atomic coordinates and isotropic or equivalent isotropic displacement parameters (Å^2^)

**Table:**

	*x*	*y*	*z*	*U*_iso_*/*U*_eq_	
Er1	0.57107 (2)	0.13041 (2)	0.482686 (17)	0.02822 (9)	
Fe1	0.29787 (7)	0.22670 (7)	0.20599 (6)	0.0353 (2)	
Fe2	0.61517 (7)	0.50027 (7)	0.17245 (6)	0.03232 (19)	
Fe3	0.92557 (8)	−0.16874 (8)	0.16882 (7)	0.0458 (2)	
O1	0.4905 (3)	0.0430 (3)	0.3963 (3)	0.0332 (8)	
O2	0.4237 (4)	−0.0753 (3)	0.3429 (3)	0.0416 (9)	
O3	0.7223 (4)	0.1034 (3)	0.3245 (3)	0.0439 (10)	
O4	0.7460 (3)	−0.0520 (3)	0.4395 (3)	0.0347 (8)	
O5	0.5175 (3)	0.3222 (3)	0.4150 (3)	0.0350 (9)	
O6	0.6368 (4)	0.4088 (3)	0.4426 (3)	0.0461 (10)	
O7	0.3589 (3)	0.2190 (3)	0.5529 (3)	0.0417 (9)	
H7C	0.3170	0.1763	0.5551	0.050*	
H7D	0.3109	0.2932	0.5512	0.050*	
O8	0.7143 (4)	0.1897 (4)	0.5211 (3)	0.0483 (11)	
H8A	0.7011	0.2610	0.5029	0.072*	
O9	0.2370 (4)	0.4566 (4)	0.5465 (5)	0.0879 (17)	
H9	0.2816	0.4923	0.5479	0.132*	
C1	0.4567 (5)	0.0134 (5)	0.3250 (4)	0.0302 (12)	
C2	0.4530 (5)	0.0820 (5)	0.2245 (4)	0.0321 (12)	
C3	0.4035 (5)	0.0641 (5)	0.1441 (4)	0.0404 (14)	
H3	0.3764	0.0023	0.1442	0.048*	
C4	0.4034 (6)	0.1561 (6)	0.0653 (4)	0.0495 (16)	
H4	0.3764	0.1656	0.0040	0.059*	
C5	0.4506 (5)	0.2312 (6)	0.0947 (4)	0.0485 (16)	
H5	0.4604	0.2987	0.0553	0.058*	
C6	0.4816 (5)	0.1893 (5)	0.1937 (4)	0.0381 (14)	
H6	0.5136	0.2238	0.2309	0.046*	
C7	0.1720 (6)	0.2378 (6)	0.3435 (5)	0.0549 (18)	
H7	0.1840	0.1835	0.4012	0.066*	
C8	0.2005 (6)	0.3414 (6)	0.3227 (5)	0.0581 (18)	
H8	0.2348	0.3679	0.3643	0.070*	
C9	0.1680 (6)	0.3987 (6)	0.2275 (5)	0.0545 (17)	
H9A	0.1768	0.4696	0.1954	0.065*	
C10	0.1203 (6)	0.3293 (7)	0.1905 (5)	0.0588 (19)	
H10	0.0922	0.3461	0.1289	0.071*	
C11	0.1219 (6)	0.2302 (7)	0.2618 (5)	0.0581 (18)	
H11	0.0947	0.1703	0.2560	0.070*	
C12	0.7856 (5)	−0.0034 (5)	0.3533 (4)	0.0353 (13)	
C13	0.9067 (5)	−0.0722 (6)	0.2873 (5)	0.0462 (15)	
C14	0.9833 (6)	−0.1957 (6)	0.3047 (6)	0.0601 (19)	
H14	0.9683	−0.2474	0.3615	0.072*	
C15	1.0859 (6)	−0.2247 (9)	0.2197 (7)	0.088 (3)	
H15	1.1510	−0.2996	0.2106	0.106*	
C16	1.0736 (8)	−0.1209 (9)	0.1505 (8)	0.092 (3)	
H16	1.1297	−0.1160	0.0887	0.110*	
C17	0.9635 (7)	−0.0273 (7)	0.1905 (6)	0.072 (2)	
H17	0.9325	0.0504	0.1597	0.087*	
C18	0.7570 (7)	−0.1674 (10)	0.1690 (7)	0.088 (3)	
H18	0.6846	−0.1330	0.2185	0.105*	
C19	0.8381 (9)	−0.2823 (9)	0.1744 (6)	0.079 (2)	
H19	0.8302	−0.3397	0.2282	0.094*	
C20	0.9348 (7)	−0.3021 (8)	0.0883 (7)	0.079 (3)	
H20	1.0029	−0.3741	0.0738	0.095*	
C21	0.9119 (11)	−0.1960 (12)	0.0280 (6)	0.102 (4)	
H21	0.9616	−0.1822	−0.0350	0.122*	
C22	0.7968 (12)	−0.1097 (9)	0.0804 (9)	0.107 (4)	
H22	0.7573	−0.0297	0.0577	0.128*	
C23	0.5589 (5)	0.4066 (5)	0.3975 (4)	0.0310 (12)	
C24	0.5130 (5)	0.5054 (5)	0.3201 (4)	0.0322 (12)	
C25	0.5456 (5)	0.6089 (5)	0.2942 (4)	0.0380 (13)	
H25	0.5915	0.6303	0.3283	0.046*	
C26	0.4961 (6)	0.6729 (5)	0.2078 (4)	0.0463 (15)	
H26	0.5043	0.7437	0.1747	0.056*	
C27	0.4318 (5)	0.6115 (5)	0.1798 (5)	0.0458 (15)	
H27	0.3903	0.6348	0.1254	0.055*	
C28	0.4417 (5)	0.5089 (5)	0.2491 (4)	0.0398 (14)	
H28	0.4072	0.4528	0.2485	0.048*	
C29	0.7650 (6)	0.3408 (6)	0.1665 (5)	0.0549 (17)	
H29	0.7790	0.2808	0.2202	0.066*	
C30	0.7031 (6)	0.3494 (6)	0.0885 (5)	0.0536 (17)	
H30	0.6686	0.2959	0.0810	0.064*	
C31	0.7022 (6)	0.4534 (6)	0.0234 (4)	0.0515 (16)	
H31	0.6667	0.4805	−0.0346	0.062*	
C32	0.7636 (5)	0.5088 (6)	0.0609 (5)	0.0478 (16)	
H32	0.7764	0.5790	0.0322	0.057*	
C33	0.8025 (5)	0.4399 (6)	0.1491 (5)	0.0494 (16)	
H33	0.8457	0.4565	0.1894	0.059*	
C34	0.8014 (8)	0.1469 (8)	0.5851 (7)	0.100 (3)	
H34A	0.7913	0.0792	0.6293	0.150*	
H34B	0.7883	0.2102	0.6272	0.150*	
H34C	0.8839	0.1221	0.5423	0.150*	
C35	0.1210 (7)	0.5346 (8)	0.5549 (8)	0.093 (3)	
H35A	0.1003	0.5868	0.6097	0.140*	
H35B	0.0662	0.4916	0.5706	0.140*	
H35C	0.1120	0.5822	0.4903	0.140*	

### Atomic displacement parameters (Å^2^)

**Table:**

	*U*^11^	*U*^22^	*U*^33^	*U*^12^	*U*^13^	*U*^23^
Er1	0.03273 (15)	0.02276 (13)	0.03056 (14)	−0.01187 (10)	−0.00674 (10)	−0.00209 (9)
Fe1	0.0327 (4)	0.0373 (5)	0.0313 (4)	−0.0084 (4)	−0.0092 (4)	0.0010 (4)
Fe2	0.0338 (4)	0.0277 (4)	0.0331 (4)	−0.0112 (3)	−0.0039 (3)	−0.0023 (3)
Fe3	0.0375 (5)	0.0441 (5)	0.0517 (5)	−0.0144 (4)	0.0043 (4)	−0.0181 (4)
O1	0.037 (2)	0.032 (2)	0.034 (2)	−0.0113 (17)	−0.0151 (17)	−0.0020 (16)
O2	0.060 (3)	0.038 (2)	0.037 (2)	−0.028 (2)	−0.0164 (19)	0.0040 (18)
O3	0.046 (2)	0.031 (2)	0.045 (2)	−0.0129 (19)	0.0053 (19)	−0.0032 (18)
O4	0.037 (2)	0.028 (2)	0.037 (2)	−0.0130 (17)	−0.0046 (17)	−0.0012 (17)
O5	0.037 (2)	0.026 (2)	0.043 (2)	−0.0159 (17)	−0.0060 (17)	0.0015 (17)
O6	0.060 (3)	0.035 (2)	0.051 (2)	−0.024 (2)	−0.019 (2)	0.0042 (19)
O7	0.035 (2)	0.027 (2)	0.059 (2)	−0.0125 (17)	−0.0017 (19)	−0.0025 (18)
O8	0.056 (3)	0.043 (2)	0.063 (3)	−0.029 (2)	−0.035 (2)	0.012 (2)
O9	0.051 (3)	0.035 (3)	0.183 (5)	−0.014 (2)	−0.034 (3)	−0.013 (3)
C1	0.025 (3)	0.031 (3)	0.032 (3)	−0.007 (2)	−0.007 (2)	−0.002 (2)
C2	0.032 (3)	0.034 (3)	0.031 (3)	−0.013 (2)	−0.006 (2)	−0.001 (2)
C3	0.044 (3)	0.041 (3)	0.032 (3)	−0.011 (3)	−0.005 (3)	−0.011 (3)
C4	0.050 (4)	0.065 (4)	0.022 (3)	−0.009 (3)	−0.011 (3)	−0.001 (3)
C5	0.043 (4)	0.054 (4)	0.039 (3)	−0.017 (3)	−0.001 (3)	0.009 (3)
C6	0.025 (3)	0.052 (4)	0.031 (3)	−0.012 (3)	−0.007 (2)	0.009 (3)
C7	0.040 (4)	0.065 (5)	0.037 (3)	−0.007 (3)	0.001 (3)	0.010 (3)
C8	0.049 (4)	0.064 (5)	0.053 (4)	−0.009 (4)	−0.003 (3)	−0.025 (4)
C9	0.052 (4)	0.038 (4)	0.051 (4)	−0.001 (3)	−0.004 (3)	0.000 (3)
C10	0.035 (4)	0.075 (5)	0.052 (4)	−0.003 (3)	−0.016 (3)	−0.002 (4)
C11	0.037 (4)	0.066 (5)	0.065 (4)	−0.017 (3)	−0.005 (3)	−0.006 (4)
C12	0.033 (3)	0.030 (3)	0.050 (4)	−0.016 (3)	−0.009 (3)	−0.011 (3)
C13	0.038 (3)	0.046 (4)	0.062 (4)	−0.023 (3)	−0.003 (3)	−0.017 (3)
C14	0.041 (4)	0.060 (5)	0.081 (5)	−0.004 (3)	−0.024 (4)	−0.028 (4)
C15	0.024 (4)	0.106 (7)	0.131 (8)	−0.003 (4)	−0.007 (4)	−0.072 (6)
C16	0.067 (5)	0.095 (6)	0.117 (6)	−0.049 (5)	0.026 (5)	−0.043 (5)
C17	0.063 (5)	0.063 (5)	0.091 (6)	−0.040 (4)	0.027 (4)	−0.034 (4)
C18	0.044 (5)	0.127 (9)	0.087 (7)	−0.012 (5)	−0.009 (5)	−0.058 (6)
C19	0.099 (7)	0.093 (7)	0.075 (6)	−0.066 (6)	−0.018 (5)	−0.011 (5)
C20	0.057 (5)	0.079 (6)	0.105 (7)	−0.020 (4)	0.000 (5)	−0.061 (6)
C21	0.133 (9)	0.163 (11)	0.043 (5)	−0.102 (9)	0.007 (5)	−0.019 (6)
C22	0.139 (10)	0.077 (7)	0.114 (8)	−0.019 (7)	−0.088 (8)	0.009 (6)
C23	0.032 (3)	0.024 (3)	0.031 (3)	−0.009 (2)	0.001 (2)	−0.007 (2)
C24	0.034 (3)	0.022 (3)	0.035 (3)	−0.009 (2)	−0.002 (2)	−0.004 (2)
C25	0.050 (4)	0.026 (3)	0.039 (3)	−0.018 (3)	−0.004 (3)	−0.007 (2)
C26	0.055 (4)	0.022 (3)	0.045 (4)	−0.005 (3)	−0.002 (3)	0.004 (3)
C27	0.040 (3)	0.040 (4)	0.052 (4)	−0.010 (3)	−0.014 (3)	0.006 (3)
C28	0.026 (3)	0.034 (3)	0.055 (4)	−0.008 (3)	−0.006 (3)	−0.004 (3)
C29	0.052 (4)	0.035 (4)	0.056 (4)	−0.005 (3)	0.001 (3)	0.007 (3)
C30	0.054 (4)	0.044 (4)	0.061 (4)	−0.023 (3)	0.009 (3)	−0.020 (3)
C31	0.056 (4)	0.059 (4)	0.031 (3)	−0.015 (3)	−0.004 (3)	−0.007 (3)
C32	0.042 (4)	0.047 (4)	0.046 (4)	−0.020 (3)	0.011 (3)	−0.005 (3)
C33	0.030 (3)	0.057 (4)	0.056 (4)	−0.013 (3)	−0.004 (3)	−0.010 (3)
C34	0.096 (7)	0.098 (7)	0.143 (8)	−0.053 (6)	−0.086 (7)	0.036 (6)
C35	0.053 (5)	0.077 (6)	0.148 (9)	−0.008 (4)	−0.036 (5)	−0.028 (6)

### Geometric parameters (Å, °)

**Table:**

Er1—O5	2.247 (3)	C4—H4	0.9300
Er1—O1	2.280 (3)	C5—C6	1.423 (7)
Er1—O8	2.311 (3)	C5—H5	0.9300
Er1—O2^i^	2.323 (3)	C6—H6	0.9300
Er1—O7	2.329 (4)	C7—C8	1.399 (9)
Er1—O3	2.368 (4)	C7—C11	1.406 (9)
Er1—O4	2.381 (4)	C7—H7	0.9300
Er1—O1^i^	2.667 (3)	C8—C9	1.414 (8)
Er1—C12	2.733 (6)	C8—H8	0.9300
Er1—C1^i^	2.882 (5)	C9—C10	1.397 (9)
Fe1—C2	2.021 (5)	C9—H9A	0.9300
Fe1—C3	2.032 (6)	C10—C11	1.403 (9)
Fe1—C7	2.035 (6)	C10—H10	0.9300
Fe1—C8	2.037 (6)	C11—H11	0.9300
Fe1—C6	2.040 (5)	C12—C13	1.477 (8)
Fe1—C10	2.041 (6)	C13—C14	1.424 (9)
Fe1—C11	2.042 (6)	C13—C17	1.430 (9)
Fe1—C9	2.048 (6)	C14—C15	1.407 (10)
Fe1—C5	2.055 (6)	C14—H14	0.9300
Fe1—C4	2.062 (6)	C15—C16	1.415 (12)
Fe2—C24	2.025 (5)	C15—H15	0.9300
Fe2—C33	2.026 (6)	C16—C17	1.392 (11)
Fe2—C32	2.034 (5)	C16—H16	0.9300
Fe2—C25	2.036 (5)	C17—H17	0.9300
Fe2—C29	2.038 (6)	C18—C19	1.342 (12)
Fe2—C31	2.039 (6)	C18—C22	1.350 (13)
Fe2—C28	2.043 (5)	C18—H18	0.9300
Fe2—C30	2.046 (6)	C19—C20	1.377 (10)
Fe2—C26	2.048 (6)	C19—H19	0.9300
Fe2—C27	2.058 (6)	C20—C21	1.368 (12)
Fe3—C21	2.014 (8)	C20—H20	0.9300
Fe3—C17	2.022 (7)	C21—C22	1.435 (14)
Fe3—C22	2.024 (8)	C21—H21	0.9300
Fe3—C13	2.025 (6)	C22—H22	0.9300
Fe3—C18	2.026 (8)	C23—C24	1.472 (7)
Fe3—C19	2.027 (7)	C24—C28	1.420 (7)
Fe3—C20	2.030 (7)	C24—C25	1.427 (7)
Fe3—C15	2.033 (7)	C25—C26	1.406 (8)
Fe3—C16	2.036 (8)	C25—H25	0.9300
Fe3—C14	2.037 (7)	C26—C27	1.414 (8)
O1—C1	1.275 (6)	C26—H26	0.9300
O1—Er1^i^	2.667 (3)	C27—C28	1.407 (8)
O2—C1	1.259 (6)	C27—H27	0.9300
O2—Er1^i^	2.323 (3)	C28—H28	0.9300
O3—C12	1.264 (6)	C29—C30	1.399 (9)
O4—C12	1.272 (6)	C29—C33	1.412 (9)
O5—C23	1.274 (6)	C29—H29	0.9300
O6—C23	1.250 (6)	C30—C31	1.408 (8)
O7—H7C	0.8500	C30—H30	0.9300
O7—H7D	0.8500	C31—C32	1.397 (8)
O8—C34	1.408 (7)	C31—H31	0.9300
O8—H8A	0.8200	C32—C33	1.397 (8)
O9—C35	1.322 (8)	C32—H32	0.9300
O9—H9	0.8200	C33—H33	0.9300
C1—C2	1.458 (7)	C34—H34A	0.9600
C1—Er1^i^	2.882 (5)	C34—H34B	0.9600
C2—C3	1.436 (7)	C34—H34C	0.9600
C2—C6	1.448 (7)	C35—H35A	0.9600
C3—C4	1.403 (8)	C35—H35B	0.9600
C3—H3	0.9300	C35—H35C	0.9600
C4—C5	1.398 (9)		
			
O5—Er1—O1	104.57 (12)	C3—C2—C1	125.2 (5)
O5—Er1—O8	77.50 (13)	C6—C2—C1	126.6 (5)
O1—Er1—O8	158.94 (14)	C3—C2—Fe1	69.7 (3)
O5—Er1—O2^i^	123.70 (13)	C6—C2—Fe1	69.8 (3)
O1—Er1—O2^i^	120.32 (12)	C1—C2—Fe1	119.6 (4)
O8—Er1—O2^i^	72.22 (13)	C4—C3—C2	108.0 (5)
O5—Er1—O7	76.11 (12)	C4—C3—Fe1	71.1 (3)
O1—Er1—O7	77.82 (13)	C2—C3—Fe1	68.8 (3)
O8—Er1—O7	122.42 (14)	C4—C3—H3	126.0
O2^i^—Er1—O7	81.74 (14)	C2—C3—H3	126.0
O5—Er1—O3	79.75 (13)	Fe1—C3—H3	125.6
O1—Er1—O3	81.98 (13)	C5—C4—C3	108.4 (5)
O8—Er1—O3	77.76 (14)	C5—C4—Fe1	69.9 (3)
O2^i^—Er1—O3	135.01 (14)	C3—C4—Fe1	68.8 (3)
O7—Er1—O3	143.23 (14)	C5—C4—H4	125.8
O5—Er1—O4	133.97 (12)	C3—C4—H4	125.8
O1—Er1—O4	80.08 (12)	Fe1—C4—H4	127.1
O8—Er1—O4	83.67 (13)	C4—C5—C6	110.0 (5)
O2^i^—Er1—O4	88.41 (13)	C4—C5—Fe1	70.4 (3)
O7—Er1—O4	146.75 (12)	C6—C5—Fe1	69.1 (3)
O3—Er1—O4	55.19 (12)	C4—C5—H5	125.0
O5—Er1—O1^i^	150.15 (12)	C6—C5—H5	125.0
O1—Er1—O1^i^	69.17 (12)	Fe1—C5—H5	127.1
O8—Er1—O1^i^	119.20 (12)	C5—C6—C2	105.8 (5)
O2^i^—Er1—O1^i^	51.34 (11)	C5—C6—Fe1	70.2 (3)
O7—Er1—O1^i^	74.04 (12)	C2—C6—Fe1	68.4 (3)
O3—Er1—O1^i^	126.00 (12)	C5—C6—H6	127.1
O4—Er1—O1^i^	75.02 (11)	C2—C6—H6	127.1
O5—Er1—C12	106.63 (15)	Fe1—C6—H6	125.8
O1—Er1—C12	81.03 (14)	C8—C7—C11	108.1 (6)
O8—Er1—C12	78.36 (15)	C8—C7—Fe1	70.0 (4)
O2^i^—Er1—C12	112.03 (16)	C11—C7—Fe1	70.1 (4)
O7—Er1—C12	158.63 (14)	C8—C7—H7	126.0
O3—Er1—C12	27.52 (14)	C11—C7—H7	126.0
O4—Er1—C12	27.72 (14)	Fe1—C7—H7	125.5
O1^i^—Er1—C12	101.23 (14)	C7—C8—C9	108.1 (6)
O5—Er1—C1^i^	142.66 (14)	C7—C8—Fe1	69.8 (4)
O1—Er1—C1^i^	95.34 (13)	C9—C8—Fe1	70.2 (4)
O8—Er1—C1^i^	94.91 (14)	C7—C8—H8	126.0
O2^i^—Er1—C1^i^	25.18 (13)	C9—C8—H8	126.0
O7—Er1—C1^i^	77.73 (13)	Fe1—C8—H8	125.6
O3—Er1—C1^i^	134.95 (14)	C10—C9—C8	107.5 (6)
O4—Er1—C1^i^	79.96 (13)	C10—C9—Fe1	69.8 (4)
O1^i^—Er1—C1^i^	26.20 (12)	C8—C9—Fe1	69.3 (4)
C12—Er1—C1^i^	107.47 (16)	C10—C9—H9A	126.2
C2—Fe1—C3	41.5 (2)	C8—C9—H9A	126.2
C2—Fe1—C7	106.4 (2)	Fe1—C9—H9A	126.2
C3—Fe1—C7	120.4 (3)	C9—C10—C11	108.6 (6)
C2—Fe1—C8	120.1 (2)	C9—C10—Fe1	70.3 (4)
C3—Fe1—C8	155.7 (3)	C11—C10—Fe1	69.9 (4)
C7—Fe1—C8	40.2 (3)	C9—C10—H10	125.7
C2—Fe1—C6	41.8 (2)	C11—C10—H10	125.7
C3—Fe1—C6	69.8 (2)	Fe1—C10—H10	125.7
C7—Fe1—C6	124.3 (2)	C10—C11—C7	107.7 (6)
C8—Fe1—C6	107.2 (3)	C10—C11—Fe1	69.9 (4)
C2—Fe1—C10	161.3 (3)	C7—C11—Fe1	69.5 (4)
C3—Fe1—C10	124.8 (3)	C10—C11—H11	126.1
C7—Fe1—C10	67.6 (3)	C7—C11—H11	126.1
C8—Fe1—C10	67.5 (3)	Fe1—C11—H11	126.0
C6—Fe1—C10	156.2 (3)	O3—C12—O4	120.3 (5)
C2—Fe1—C11	124.0 (3)	O3—C12—C13	120.1 (5)
C3—Fe1—C11	107.2 (3)	O4—C12—C13	119.6 (5)
C7—Fe1—C11	40.3 (3)	O3—C12—Er1	59.9 (3)
C8—Fe1—C11	67.6 (3)	O4—C12—Er1	60.5 (3)
C6—Fe1—C11	161.4 (2)	C13—C12—Er1	175.5 (4)
C10—Fe1—C11	40.2 (3)	C14—C13—C17	108.1 (6)
C2—Fe1—C9	156.1 (3)	C14—C13—C12	126.6 (6)
C3—Fe1—C9	161.7 (2)	C17—C13—C12	125.2 (6)
C7—Fe1—C9	67.8 (3)	C14—C13—Fe3	69.9 (4)
C8—Fe1—C9	40.5 (2)	C17—C13—Fe3	69.2 (4)
C6—Fe1—C9	121.0 (3)	C12—C13—Fe3	123.5 (4)
C10—Fe1—C9	39.9 (3)	C15—C14—C13	107.0 (7)
C11—Fe1—C9	67.5 (3)	C15—C14—Fe3	69.6 (4)
C2—Fe1—C5	68.3 (2)	C13—C14—Fe3	69.0 (4)
C3—Fe1—C5	67.5 (2)	C15—C14—H14	126.5
C7—Fe1—C5	162.4 (3)	C13—C14—H14	126.5
C8—Fe1—C5	126.7 (3)	Fe3—C14—H14	126.4
C6—Fe1—C5	40.7 (2)	C14—C15—C16	108.7 (7)
C10—Fe1—C5	122.6 (3)	C14—C15—Fe3	69.9 (4)
C11—Fe1—C5	156.5 (3)	C16—C15—Fe3	69.8 (5)
C9—Fe1—C5	109.8 (3)	C14—C15—H15	125.7
C2—Fe1—C4	68.5 (2)	C16—C15—H15	125.7
C3—Fe1—C4	40.1 (2)	Fe3—C15—H15	126.2
C7—Fe1—C4	155.8 (3)	C17—C16—C15	108.6 (7)
C8—Fe1—C4	163.0 (3)	C17—C16—Fe3	69.4 (4)
C6—Fe1—C4	68.6 (2)	C15—C16—Fe3	69.5 (4)
C10—Fe1—C4	109.3 (3)	C17—C16—H16	125.7
C11—Fe1—C4	121.6 (3)	C15—C16—H16	125.7
C9—Fe1—C4	126.4 (2)	Fe3—C16—H16	126.9
C5—Fe1—C4	39.7 (2)	C16—C17—C13	107.6 (7)
C24—Fe2—C33	119.1 (2)	C16—C17—Fe3	70.5 (4)
C24—Fe2—C32	154.4 (2)	C13—C17—Fe3	69.4 (4)
C33—Fe2—C32	40.3 (2)	C16—C17—H17	126.2
C24—Fe2—C25	41.1 (2)	C13—C17—H17	126.2
C33—Fe2—C25	106.1 (2)	Fe3—C17—H17	125.5
C32—Fe2—C25	119.9 (2)	C19—C18—C22	109.4 (9)
C24—Fe2—C29	106.2 (2)	C19—C18—Fe3	70.7 (5)
C33—Fe2—C29	40.7 (2)	C22—C18—Fe3	70.5 (5)
C32—Fe2—C29	68.0 (2)	C19—C18—H18	125.3
C25—Fe2—C29	123.8 (3)	C22—C18—H18	125.3
C24—Fe2—C31	162.8 (2)	Fe3—C18—H18	125.1
C33—Fe2—C31	67.5 (3)	C18—C19—C20	109.7 (8)
C32—Fe2—C31	40.1 (2)	C18—C19—Fe3	70.6 (5)
C25—Fe2—C31	155.7 (2)	C20—C19—Fe3	70.3 (4)
C29—Fe2—C31	67.7 (3)	C18—C19—H19	125.1
C24—Fe2—C28	40.9 (2)	C20—C19—H19	125.1
C33—Fe2—C28	155.3 (2)	Fe3—C19—H19	125.6
C32—Fe2—C28	163.5 (2)	C21—C20—C19	107.1 (8)
C25—Fe2—C28	68.4 (2)	C21—C20—Fe3	69.6 (5)
C29—Fe2—C28	121.0 (2)	C19—C20—Fe3	70.0 (4)
C31—Fe2—C28	127.2 (3)	C21—C20—H20	126.4
C24—Fe2—C30	124.8 (2)	C19—C20—H20	126.4
C33—Fe2—C30	67.7 (3)	Fe3—C20—H20	125.5
C32—Fe2—C30	67.7 (2)	C20—C21—C22	107.2 (8)
C25—Fe2—C30	161.1 (3)	C20—C21—Fe3	70.9 (5)
C29—Fe2—C30	40.1 (3)	C22—C21—Fe3	69.6 (5)
C31—Fe2—C30	40.3 (2)	C20—C21—H21	126.4
C28—Fe2—C30	109.4 (2)	C22—C21—H21	126.4
C24—Fe2—C26	68.5 (2)	Fe3—C21—H21	124.8
C33—Fe2—C26	124.5 (3)	C18—C22—C21	106.6 (9)
C32—Fe2—C26	108.4 (2)	C18—C22—Fe3	70.6 (5)
C25—Fe2—C26	40.3 (2)	C21—C22—Fe3	68.8 (5)
C29—Fe2—C26	160.8 (3)	C18—C22—H22	126.7
C31—Fe2—C26	122.6 (2)	C21—C22—H22	126.7
C28—Fe2—C26	67.8 (2)	Fe3—C22—H22	125.5
C30—Fe2—C26	157.8 (3)	O6—C23—O5	123.0 (5)
C24—Fe2—C27	68.4 (2)	O6—C23—C24	118.8 (5)
C33—Fe2—C27	162.1 (3)	O5—C23—C24	118.2 (5)
C32—Fe2—C27	126.7 (2)	C28—C24—C25	107.2 (5)
C25—Fe2—C27	68.0 (2)	C28—C24—C23	126.9 (5)
C29—Fe2—C27	156.7 (3)	C25—C24—C23	125.5 (5)
C31—Fe2—C27	110.5 (3)	C28—C24—Fe2	70.2 (3)
C28—Fe2—C27	40.1 (2)	C25—C24—Fe2	69.8 (3)
C30—Fe2—C27	123.2 (3)	C23—C24—Fe2	119.5 (3)
C26—Fe2—C27	40.3 (2)	C26—C25—C24	107.9 (5)
C21—Fe3—C17	121.4 (4)	C26—C25—Fe2	70.3 (3)
C21—Fe3—C22	41.6 (4)	C24—C25—Fe2	69.0 (3)
C17—Fe3—C22	108.7 (4)	C26—C25—H25	126.0
C21—Fe3—C13	156.8 (4)	C24—C25—H25	126.0
C17—Fe3—C13	41.4 (3)	Fe2—C25—H25	126.2
C22—Fe3—C13	120.5 (4)	C25—C26—C27	108.5 (5)
C21—Fe3—C18	67.1 (4)	C25—C26—Fe2	69.4 (3)
C17—Fe3—C18	126.9 (4)	C27—C26—Fe2	70.2 (3)
C22—Fe3—C18	38.9 (4)	C25—C26—H26	125.7
C13—Fe3—C18	108.6 (3)	C27—C26—H26	125.7
C21—Fe3—C19	66.3 (3)	Fe2—C26—H26	126.2
C17—Fe3—C19	162.8 (4)	C28—C27—C26	107.8 (5)
C22—Fe3—C19	65.7 (4)	C28—C27—Fe2	69.4 (3)
C13—Fe3—C19	125.7 (3)	C26—C27—Fe2	69.5 (3)
C18—Fe3—C19	38.7 (3)	C28—C27—H27	126.1
C21—Fe3—C20	39.5 (4)	C26—C27—H27	126.1
C17—Fe3—C20	155.6 (3)	Fe2—C27—H27	126.7
C22—Fe3—C20	67.6 (4)	C27—C28—C24	108.5 (5)
C13—Fe3—C20	161.9 (4)	C27—C28—Fe2	70.5 (3)
C18—Fe3—C20	66.5 (3)	C24—C28—Fe2	68.9 (3)
C19—Fe3—C20	39.7 (3)	C27—C28—H28	125.7
C21—Fe3—C15	125.7 (4)	C24—C28—H28	125.7
C17—Fe3—C15	68.4 (3)	Fe2—C28—H28	126.4
C22—Fe3—C15	164.4 (5)	C30—C29—C33	107.6 (6)
C13—Fe3—C15	68.2 (3)	C30—C29—Fe2	70.3 (4)
C18—Fe3—C15	154.8 (4)	C33—C29—Fe2	69.2 (3)
C19—Fe3—C15	121.4 (4)	C30—C29—H29	126.2
C20—Fe3—C15	108.2 (3)	C33—C29—H29	126.2
C21—Fe3—C16	108.9 (4)	Fe2—C29—H29	125.9
C17—Fe3—C16	40.1 (3)	C29—C30—C31	108.0 (6)
C22—Fe3—C16	127.4 (5)	C29—C30—Fe2	69.7 (4)
C13—Fe3—C16	68.2 (3)	C31—C30—Fe2	69.6 (4)
C18—Fe3—C16	163.5 (4)	C29—C30—H30	126.0
C19—Fe3—C16	156.2 (4)	C31—C30—H30	126.0
C20—Fe3—C16	121.5 (3)	Fe2—C30—H30	126.3
C15—Fe3—C16	40.7 (3)	C32—C31—C30	108.3 (6)
C21—Fe3—C14	161.4 (4)	C32—C31—Fe2	69.7 (3)
C17—Fe3—C14	69.4 (3)	C30—C31—Fe2	70.1 (3)
C22—Fe3—C14	154.4 (4)	C32—C31—H31	125.9
C13—Fe3—C14	41.0 (2)	C30—C31—H31	125.9
C18—Fe3—C14	120.5 (4)	Fe2—C31—H31	125.9
C19—Fe3—C14	108.1 (3)	C31—C32—C33	107.8 (6)
C20—Fe3—C14	124.8 (4)	C31—C32—Fe2	70.1 (3)
C15—Fe3—C14	40.5 (3)	C33—C32—Fe2	69.5 (3)
C16—Fe3—C14	68.5 (3)	C31—C32—H32	126.1
C1—O1—Er1	162.6 (3)	C33—C32—H32	126.1
C1—O1—Er1^i^	86.3 (3)	Fe2—C32—H32	125.8
Er1—O1—Er1^i^	110.83 (12)	C32—C33—C29	108.4 (6)
C1—O2—Er1^i^	103.1 (3)	C32—C33—Fe2	70.2 (3)
C12—O3—Er1	92.6 (3)	C29—C33—Fe2	70.1 (3)
C12—O4—Er1	91.8 (3)	C32—C33—H33	125.8
C23—O5—Er1	138.8 (3)	C29—C33—H33	125.8
Er1—O7—H7C	115.9	Fe2—C33—H33	125.4
Er1—O7—H7D	129.0	O8—C34—H34A	109.5
H7C—O7—H7D	108.7	O8—C34—H34B	109.5
C34—O8—Er1	137.5 (4)	H34A—C34—H34B	109.5
C34—O8—H8A	109.5	O8—C34—H34C	109.5
Er1—O8—H8A	111.4	H34A—C34—H34C	109.5
C35—O9—H9	109.5	H34B—C34—H34C	109.5
O2—C1—O1	119.0 (4)	O9—C35—H35A	109.5
O2—C1—C2	119.7 (5)	O9—C35—H35B	109.5
O1—C1—C2	121.2 (5)	H35A—C35—H35B	109.5
O2—C1—Er1^i^	51.7 (2)	O9—C35—H35C	109.5
O1—C1—Er1^i^	67.5 (3)	H35A—C35—H35C	109.5
C2—C1—Er1^i^	170.1 (4)	H35B—C35—H35C	109.5
C3—C2—C6	107.7 (5)		
			
O5—Er1—O1—C1	39.5 (11)	C18—Fe3—C15—C14	−49.3 (9)
O8—Er1—O1—C1	−53.5 (13)	C19—Fe3—C15—C14	−81.1 (5)
O2^i^—Er1—O1—C1	−175.8 (11)	C20—Fe3—C15—C14	−122.7 (5)
O7—Er1—O1—C1	111.5 (11)	C16—Fe3—C15—C14	119.9 (7)
O3—Er1—O1—C1	−37.6 (11)	C21—Fe3—C15—C16	77.2 (7)
O4—Er1—O1—C1	−93.5 (11)	C17—Fe3—C15—C16	−36.8 (5)
O1^i^—Er1—O1—C1	−171.2 (12)	C22—Fe3—C15—C16	45.6 (15)
C12—Er1—O1—C1	−65.4 (11)	C13—Fe3—C15—C16	−81.5 (5)
C1^i^—Er1—O1—C1	−172.3 (10)	C18—Fe3—C15—C16	−169.2 (7)
O5—Er1—O1—Er1^i^	−149.31 (13)	C19—Fe3—C15—C16	159.0 (5)
O8—Er1—O1—Er1^i^	117.7 (3)	C20—Fe3—C15—C16	117.4 (5)
O2^i^—Er1—O1—Er1^i^	−4.6 (2)	C14—Fe3—C15—C16	−119.9 (7)
O7—Er1—O1—Er1^i^	−77.36 (14)	C14—C15—C16—C17	−0.7 (9)
O3—Er1—O1—Er1^i^	133.54 (15)	Fe3—C15—C16—C17	58.6 (6)
O4—Er1—O1—Er1^i^	77.63 (14)	C14—C15—C16—Fe3	−59.3 (5)
O1^i^—Er1—O1—Er1^i^	0.0	C21—Fe3—C16—C17	116.6 (7)
C12—Er1—O1—Er1^i^	105.72 (17)	C22—Fe3—C16—C17	73.7 (7)
C1^i^—Er1—O1—Er1^i^	−1.17 (16)	C13—Fe3—C16—C17	−38.7 (5)
O5—Er1—O3—C12	167.5 (3)	C18—Fe3—C16—C17	43.4 (15)
O1—Er1—O3—C12	−86.0 (3)	C19—Fe3—C16—C17	−169.4 (8)
O8—Er1—O3—C12	88.3 (3)	C20—Fe3—C16—C17	158.4 (6)
O2^i^—Er1—O3—C12	39.5 (4)	C15—Fe3—C16—C17	−120.2 (8)
O7—Er1—O3—C12	−143.0 (3)	C14—Fe3—C16—C17	−83.0 (5)
O4—Er1—O3—C12	−2.5 (3)	C21—Fe3—C16—C15	−123.1 (6)
O1^i^—Er1—O3—C12	−29.1 (4)	C17—Fe3—C16—C15	120.2 (8)
C1^i^—Er1—O3—C12	3.6 (4)	C22—Fe3—C16—C15	−166.0 (6)
O5—Er1—O4—C12	−11.1 (4)	C13—Fe3—C16—C15	81.5 (5)
O1—Er1—O4—C12	89.6 (3)	C18—Fe3—C16—C15	163.7 (10)
O8—Er1—O4—C12	−77.0 (3)	C19—Fe3—C16—C15	−49.2 (11)
O2^i^—Er1—O4—C12	−149.3 (3)	C20—Fe3—C16—C15	−81.4 (6)
O7—Er1—O4—C12	138.5 (3)	C14—Fe3—C16—C15	37.2 (4)
O3—Er1—O4—C12	2.5 (3)	C15—C16—C17—C13	1.1 (9)
O1^i^—Er1—O4—C12	160.5 (3)	Fe3—C16—C17—C13	59.7 (5)
C1^i^—Er1—O4—C12	−173.1 (3)	C15—C16—C17—Fe3	−58.6 (6)
O1—Er1—O5—C23	−150.0 (5)	C14—C13—C17—C16	−1.0 (8)
O8—Er1—O5—C23	8.4 (5)	C12—C13—C17—C16	−177.4 (6)
O2^i^—Er1—O5—C23	66.8 (5)	Fe3—C13—C17—C16	−60.4 (5)
O7—Er1—O5—C23	136.8 (5)	C14—C13—C17—Fe3	59.4 (4)
O3—Er1—O5—C23	−71.2 (5)	C12—C13—C17—Fe3	−117.0 (6)
O4—Er1—O5—C23	−59.8 (5)	C21—Fe3—C17—C16	−82.2 (7)
O1^i^—Er1—O5—C23	136.6 (4)	C22—Fe3—C17—C16	−126.4 (7)
C12—Er1—O5—C23	−65.2 (5)	C13—Fe3—C17—C16	118.5 (7)
C1^i^—Er1—O5—C23	90.0 (5)	C18—Fe3—C17—C16	−165.9 (6)
O5—Er1—O8—C34	166.8 (7)	C19—Fe3—C17—C16	165.5 (11)
O1—Er1—O8—C34	−95.1 (8)	C20—Fe3—C17—C16	−49.4 (11)
O2^i^—Er1—O8—C34	34.9 (7)	C15—Fe3—C17—C16	37.3 (5)
O7—Er1—O8—C34	102.4 (7)	C14—Fe3—C17—C16	80.8 (6)
O3—Er1—O8—C34	−111.2 (7)	C21—Fe3—C17—C13	159.3 (5)
O4—Er1—O8—C34	−55.5 (7)	C22—Fe3—C17—C13	115.2 (6)
O1^i^—Er1—O8—C34	13.4 (8)	C18—Fe3—C17—C13	75.7 (6)
C12—Er1—O8—C34	−83.0 (7)	C19—Fe3—C17—C13	47.0 (14)
C1^i^—Er1—O8—C34	23.8 (7)	C20—Fe3—C17—C13	−167.8 (7)
Er1^i^—O2—C1—O1	4.8 (5)	C15—Fe3—C17—C13	−81.2 (5)
Er1^i^—O2—C1—C2	−173.9 (4)	C16—Fe3—C17—C13	−118.5 (7)
Er1—O1—C1—O2	167.7 (8)	C14—Fe3—C17—C13	−37.7 (4)
Er1^i^—O1—C1—O2	−4.1 (5)	C21—Fe3—C18—C19	80.1 (6)
Er1—O1—C1—C2	−13.7 (14)	C17—Fe3—C18—C19	−166.9 (5)
Er1^i^—O1—C1—C2	174.6 (5)	C22—Fe3—C18—C19	119.9 (8)
Er1—O1—C1—Er1^i^	171.7 (11)	C13—Fe3—C18—C19	−124.4 (5)
O2—C1—C2—C3	5.9 (8)	C20—Fe3—C18—C19	36.9 (5)
O1—C1—C2—C3	−172.7 (5)	C15—Fe3—C18—C19	−46.0 (10)
Er1^i^—C1—C2—C3	−23 (3)	C16—Fe3—C18—C19	159.5 (11)
O2—C1—C2—C6	176.7 (5)	C14—Fe3—C18—C19	−80.9 (6)
O1—C1—C2—C6	−2.0 (8)	C21—Fe3—C18—C22	−39.8 (6)
Er1^i^—C1—C2—C6	147.6 (19)	C17—Fe3—C18—C22	73.2 (7)
O2—C1—C2—Fe1	90.8 (6)	C13—Fe3—C18—C22	115.7 (6)
O1—C1—C2—Fe1	−87.8 (5)	C19—Fe3—C18—C22	−119.9 (8)
Er1^i^—C1—C2—Fe1	62 (2)	C20—Fe3—C18—C22	−83.0 (6)
C7—Fe1—C2—C3	117.6 (4)	C15—Fe3—C18—C22	−165.9 (7)
C8—Fe1—C2—C3	159.0 (4)	C16—Fe3—C18—C22	39.6 (14)
C6—Fe1—C2—C3	−118.8 (4)	C14—Fe3—C18—C22	159.2 (6)
C10—Fe1—C2—C3	49.3 (8)	C22—C18—C19—C20	0.5 (10)
C11—Fe1—C2—C3	77.1 (4)	Fe3—C18—C19—C20	−59.6 (5)
C9—Fe1—C2—C3	−170.4 (5)	C22—C18—C19—Fe3	60.1 (7)
C5—Fe1—C2—C3	−80.1 (4)	C21—Fe3—C19—C18	−82.5 (6)
C4—Fe1—C2—C3	−37.3 (3)	C17—Fe3—C19—C18	37.9 (14)
C3—Fe1—C2—C6	118.8 (4)	C22—Fe3—C19—C18	−36.7 (6)
C7—Fe1—C2—C6	−123.5 (3)	C13—Fe3—C19—C18	74.4 (6)
C8—Fe1—C2—C6	−82.1 (4)	C20—Fe3—C19—C18	−120.4 (8)
C10—Fe1—C2—C6	168.1 (7)	C15—Fe3—C19—C18	159.0 (5)
C11—Fe1—C2—C6	−164.1 (3)	C16—Fe3—C19—C18	−165.7 (9)
C9—Fe1—C2—C6	−51.6 (7)	C14—Fe3—C19—C18	116.5 (6)
C5—Fe1—C2—C6	38.7 (3)	C21—Fe3—C19—C20	37.9 (6)
C4—Fe1—C2—C6	81.5 (3)	C17—Fe3—C19—C20	158.3 (11)
C3—Fe1—C2—C1	−119.8 (5)	C22—Fe3—C19—C20	83.7 (6)
C7—Fe1—C2—C1	−2.1 (5)	C13—Fe3—C19—C20	−165.2 (5)
C8—Fe1—C2—C1	39.3 (5)	C18—Fe3—C19—C20	120.4 (8)
C6—Fe1—C2—C1	121.4 (5)	C15—Fe3—C19—C20	−80.6 (6)
C10—Fe1—C2—C1	−70.4 (9)	C16—Fe3—C19—C20	−45.3 (11)
C11—Fe1—C2—C1	−42.7 (5)	C14—Fe3—C19—C20	−123.1 (6)
C9—Fe1—C2—C1	69.8 (7)	C18—C19—C20—C21	−0.2 (9)
C5—Fe1—C2—C1	160.1 (5)	Fe3—C19—C20—C21	−60.0 (6)
C4—Fe1—C2—C1	−157.1 (5)	C18—C19—C20—Fe3	59.8 (6)
C6—C2—C3—C4	0.9 (6)	C17—Fe3—C20—C21	−46.7 (11)
C1—C2—C3—C4	173.1 (5)	C22—Fe3—C20—C21	39.6 (6)
Fe1—C2—C3—C4	60.6 (4)	C13—Fe3—C20—C21	160.0 (9)
C6—C2—C3—Fe1	−59.7 (4)	C18—Fe3—C20—C21	82.0 (6)
C1—C2—C3—Fe1	112.5 (5)	C19—Fe3—C20—C21	118.0 (8)
C2—Fe1—C3—C4	−118.9 (5)	C15—Fe3—C20—C21	−124.4 (6)
C7—Fe1—C3—C4	160.9 (4)	C16—Fe3—C20—C21	−81.7 (7)
C8—Fe1—C3—C4	−167.5 (6)	C14—Fe3—C20—C21	−166.1 (6)
C6—Fe1—C3—C4	−80.4 (4)	C21—Fe3—C20—C19	−118.0 (8)
C10—Fe1—C3—C4	78.3 (4)	C17—Fe3—C20—C19	−164.7 (8)
C11—Fe1—C3—C4	118.9 (4)	C22—Fe3—C20—C19	−78.4 (6)
C9—Fe1—C3—C4	48.7 (9)	C13—Fe3—C20—C19	42.0 (12)
C5—Fe1—C3—C4	−36.7 (3)	C18—Fe3—C20—C19	−36.0 (5)
C7—Fe1—C3—C2	−80.2 (4)	C15—Fe3—C20—C19	117.6 (6)
C8—Fe1—C3—C2	−48.6 (7)	C16—Fe3—C20—C19	160.3 (6)
C6—Fe1—C3—C2	38.5 (3)	C14—Fe3—C20—C19	75.9 (6)
C10—Fe1—C3—C2	−162.8 (3)	C19—C20—C21—C22	−0.1 (9)
C11—Fe1—C3—C2	−122.2 (3)	Fe3—C20—C21—C22	−60.4 (6)
C9—Fe1—C3—C2	167.6 (7)	C19—C20—C21—Fe3	60.3 (5)
C5—Fe1—C3—C2	82.2 (3)	C17—Fe3—C21—C20	159.4 (5)
C4—Fe1—C3—C2	118.9 (5)	C22—Fe3—C21—C20	−117.5 (8)
C2—C3—C4—C5	−0.3 (7)	C13—Fe3—C21—C20	−164.4 (6)
Fe1—C3—C4—C5	58.8 (4)	C18—Fe3—C21—C20	−80.3 (6)
C2—C3—C4—Fe1	−59.2 (4)	C19—Fe3—C21—C20	−38.0 (5)
C2—Fe1—C4—C5	−81.6 (4)	C15—Fe3—C21—C20	74.7 (7)
C3—Fe1—C4—C5	−120.2 (5)	C16—Fe3—C21—C20	116.9 (6)
C7—Fe1—C4—C5	−163.7 (5)	C14—Fe3—C21—C20	38.1 (13)
C8—Fe1—C4—C5	42.1 (10)	C17—Fe3—C21—C22	−83.1 (7)
C6—Fe1—C4—C5	−36.6 (3)	C13—Fe3—C21—C22	−46.8 (10)
C10—Fe1—C4—C5	118.2 (4)	C18—Fe3—C21—C22	37.3 (6)
C11—Fe1—C4—C5	160.9 (4)	C19—Fe3—C21—C22	79.5 (6)
C9—Fe1—C4—C5	76.9 (4)	C20—Fe3—C21—C22	117.5 (8)
C2—Fe1—C4—C3	38.6 (3)	C15—Fe3—C21—C22	−167.8 (6)
C7—Fe1—C4—C3	−43.5 (7)	C16—Fe3—C21—C22	−125.6 (7)
C8—Fe1—C4—C3	162.3 (8)	C14—Fe3—C21—C22	155.7 (10)
C6—Fe1—C4—C3	83.6 (4)	C19—C18—C22—C21	−0.5 (11)
C10—Fe1—C4—C3	−121.6 (4)	Fe3—C18—C22—C21	59.7 (6)
C11—Fe1—C4—C3	−78.9 (4)	C19—C18—C22—Fe3	−60.2 (6)
C9—Fe1—C4—C3	−162.9 (4)	C20—C21—C22—C18	0.4 (10)
C5—Fe1—C4—C3	120.2 (5)	Fe3—C21—C22—C18	−60.8 (6)
C3—C4—C5—C6	−0.4 (7)	C20—C21—C22—Fe3	61.2 (6)
Fe1—C4—C5—C6	57.8 (4)	C21—Fe3—C22—C18	117.5 (9)
C3—C4—C5—Fe1	−58.1 (4)	C17—Fe3—C22—C18	−126.0 (6)
C2—Fe1—C5—C4	82.0 (4)	C13—Fe3—C22—C18	−82.1 (6)
C3—Fe1—C5—C4	37.0 (3)	C19—Fe3—C22—C18	36.5 (5)
C7—Fe1—C5—C4	157.6 (8)	C20—Fe3—C22—C18	79.8 (6)
C8—Fe1—C5—C4	−165.9 (4)	C15—Fe3—C22—C18	157.3 (12)
C6—Fe1—C5—C4	121.7 (5)	C16—Fe3—C22—C18	−166.8 (5)
C10—Fe1—C5—C4	−81.0 (4)	C14—Fe3—C22—C18	−44.8 (11)
C11—Fe1—C5—C4	−44.4 (8)	C17—Fe3—C22—C21	116.5 (6)
C9—Fe1—C5—C4	−123.5 (4)	C13—Fe3—C22—C21	160.5 (5)
C2—Fe1—C5—C6	−39.7 (3)	C18—Fe3—C22—C21	−117.5 (9)
C3—Fe1—C5—C6	−84.7 (4)	C19—Fe3—C22—C21	−81.0 (6)
C7—Fe1—C5—C6	35.9 (10)	C20—Fe3—C22—C21	−37.6 (5)
C8—Fe1—C5—C6	72.5 (5)	C15—Fe3—C22—C21	39.8 (17)
C10—Fe1—C5—C6	157.3 (4)	C16—Fe3—C22—C21	75.7 (7)
C11—Fe1—C5—C6	−166.1 (6)	C14—Fe3—C22—C21	−162.3 (7)
C9—Fe1—C5—C6	114.8 (4)	Er1—O5—C23—O6	−19.6 (8)
C4—Fe1—C5—C6	−121.7 (5)	Er1—O5—C23—C24	160.0 (3)
C4—C5—C6—C2	0.9 (7)	O6—C23—C24—C28	168.7 (5)
Fe1—C5—C6—C2	59.5 (4)	O5—C23—C24—C28	−10.9 (8)
C4—C5—C6—Fe1	−58.6 (4)	O6—C23—C24—C25	−3.0 (8)
C3—C2—C6—C5	−1.1 (6)	O5—C23—C24—C25	177.4 (5)
C1—C2—C6—C5	−173.2 (5)	O6—C23—C24—Fe2	82.2 (5)
Fe1—C2—C6—C5	−60.7 (4)	O5—C23—C24—Fe2	−97.4 (5)
C3—C2—C6—Fe1	59.6 (4)	C33—Fe2—C24—C28	−161.1 (3)
C1—C2—C6—Fe1	−112.5 (5)	C32—Fe2—C24—C28	168.2 (5)
C2—Fe1—C6—C5	116.9 (5)	C25—Fe2—C24—C28	117.8 (5)
C3—Fe1—C6—C5	78.7 (4)	C29—Fe2—C24—C28	−119.0 (4)
C7—Fe1—C6—C5	−167.6 (4)	C31—Fe2—C24—C28	−52.6 (9)
C8—Fe1—C6—C5	−126.8 (4)	C30—Fe2—C24—C28	−79.2 (4)
C10—Fe1—C6—C5	−53.7 (8)	C26—Fe2—C24—C28	80.5 (4)
C11—Fe1—C6—C5	162.5 (8)	C27—Fe2—C24—C28	37.0 (3)
C9—Fe1—C6—C5	−84.8 (4)	C33—Fe2—C24—C25	81.1 (4)
C4—Fe1—C6—C5	35.7 (4)	C32—Fe2—C24—C25	50.4 (7)
C3—Fe1—C6—C2	−38.2 (3)	C29—Fe2—C24—C25	123.2 (4)
C7—Fe1—C6—C2	75.5 (4)	C31—Fe2—C24—C25	−170.4 (7)
C8—Fe1—C6—C2	116.2 (3)	C28—Fe2—C24—C25	−117.8 (5)
C10—Fe1—C6—C2	−170.6 (6)	C30—Fe2—C24—C25	162.9 (4)
C11—Fe1—C6—C2	45.6 (10)	C26—Fe2—C24—C25	−37.4 (3)
C9—Fe1—C6—C2	158.3 (3)	C27—Fe2—C24—C25	−80.9 (4)
C5—Fe1—C6—C2	−116.9 (5)	C33—Fe2—C24—C23	−39.1 (5)
C4—Fe1—C6—C2	−81.2 (3)	C32—Fe2—C24—C23	−69.8 (7)
C2—Fe1—C7—C8	117.5 (4)	C25—Fe2—C24—C23	−120.2 (6)
C3—Fe1—C7—C8	160.4 (4)	C29—Fe2—C24—C23	3.0 (5)
C6—Fe1—C7—C8	75.3 (5)	C31—Fe2—C24—C23	69.4 (9)
C10—Fe1—C7—C8	−81.2 (4)	C28—Fe2—C24—C23	122.0 (5)
C11—Fe1—C7—C8	−118.9 (6)	C30—Fe2—C24—C23	42.7 (5)
C9—Fe1—C7—C8	−37.9 (4)	C26—Fe2—C24—C23	−157.6 (5)
C5—Fe1—C7—C8	47.8 (10)	C27—Fe2—C24—C23	159.0 (5)
C4—Fe1—C7—C8	−168.6 (5)	C28—C24—C25—C26	−0.8 (6)
C2—Fe1—C7—C11	−123.6 (4)	C23—C24—C25—C26	172.2 (5)
C3—Fe1—C7—C11	−80.7 (5)	Fe2—C24—C25—C26	59.8 (4)
C8—Fe1—C7—C11	118.9 (6)	C28—C24—C25—Fe2	−60.6 (4)
C6—Fe1—C7—C11	−165.8 (4)	C23—C24—C25—Fe2	112.5 (5)
C10—Fe1—C7—C11	37.7 (4)	C24—Fe2—C25—C26	−119.2 (5)
C9—Fe1—C7—C11	81.0 (4)	C33—Fe2—C25—C26	124.8 (4)
C5—Fe1—C7—C11	166.6 (8)	C32—Fe2—C25—C26	83.4 (4)
C4—Fe1—C7—C11	−49.8 (8)	C29—Fe2—C25—C26	165.6 (4)
C11—C7—C8—C9	0.0 (7)	C31—Fe2—C25—C26	54.0 (7)
Fe1—C7—C8—C9	60.0 (4)	C28—Fe2—C25—C26	−80.7 (4)
C11—C7—C8—Fe1	−60.0 (4)	C30—Fe2—C25—C26	−167.4 (7)
C2—Fe1—C8—C7	−79.5 (4)	C27—Fe2—C25—C26	−37.3 (3)
C3—Fe1—C8—C7	−44.4 (8)	C33—Fe2—C25—C24	−116.0 (3)
C6—Fe1—C8—C7	−123.2 (4)	C32—Fe2—C25—C24	−157.4 (3)
C10—Fe1—C8—C7	81.4 (4)	C29—Fe2—C25—C24	−75.2 (4)
C11—Fe1—C8—C7	37.8 (4)	C31—Fe2—C25—C24	173.2 (5)
C9—Fe1—C8—C7	118.9 (6)	C28—Fe2—C25—C24	38.5 (3)
C5—Fe1—C8—C7	−163.8 (4)	C30—Fe2—C25—C24	−48.2 (9)
C4—Fe1—C8—C7	164.0 (8)	C26—Fe2—C25—C24	119.2 (5)
C2—Fe1—C8—C9	161.5 (4)	C27—Fe2—C25—C24	81.9 (3)
C3—Fe1—C8—C9	−163.4 (6)	C24—C25—C26—C27	0.6 (6)
C7—Fe1—C8—C9	−118.9 (6)	Fe2—C25—C26—C27	59.5 (4)
C6—Fe1—C8—C9	117.8 (4)	C24—C25—C26—Fe2	−59.0 (4)
C10—Fe1—C8—C9	−37.5 (4)	C24—Fe2—C26—C25	38.1 (3)
C11—Fe1—C8—C9	−81.1 (4)	C33—Fe2—C26—C25	−73.1 (4)
C5—Fe1—C8—C9	77.3 (5)	C32—Fe2—C26—C25	−114.8 (4)
C4—Fe1—C8—C9	45.0 (10)	C29—Fe2—C26—C25	−39.0 (9)
C7—C8—C9—C10	−0.2 (7)	C31—Fe2—C26—C25	−156.7 (3)
Fe1—C8—C9—C10	59.6 (4)	C28—Fe2—C26—C25	82.3 (4)
C7—C8—C9—Fe1	−59.8 (4)	C30—Fe2—C26—C25	169.2 (6)
C2—Fe1—C9—C10	−161.4 (5)	C27—Fe2—C26—C25	119.7 (5)
C3—Fe1—C9—C10	39.1 (10)	C24—Fe2—C26—C27	−81.6 (4)
C7—Fe1—C9—C10	−81.2 (4)	C33—Fe2—C26—C27	167.1 (3)
C8—Fe1—C9—C10	−118.8 (6)	C32—Fe2—C26—C27	125.5 (4)
C6—Fe1—C9—C10	161.1 (4)	C25—Fe2—C26—C27	−119.7 (5)
C11—Fe1—C9—C10	−37.5 (4)	C29—Fe2—C26—C27	−158.7 (7)
C5—Fe1—C9—C10	117.5 (4)	C31—Fe2—C26—C27	83.6 (4)
C4—Fe1—C9—C10	76.1 (5)	C28—Fe2—C26—C27	−37.4 (3)
C2—Fe1—C9—C8	−42.6 (8)	C30—Fe2—C26—C27	49.5 (8)
C3—Fe1—C9—C8	158.0 (7)	C25—C26—C27—C28	−0.1 (7)
C7—Fe1—C9—C8	37.6 (4)	Fe2—C26—C27—C28	58.9 (4)
C6—Fe1—C9—C8	−80.1 (4)	C25—C26—C27—Fe2	−59.0 (4)
C10—Fe1—C9—C8	118.8 (6)	C24—Fe2—C27—C28	−37.6 (3)
C11—Fe1—C9—C8	81.4 (4)	C33—Fe2—C27—C28	−156.0 (7)
C5—Fe1—C9—C8	−123.7 (4)	C32—Fe2—C27—C28	166.3 (4)
C4—Fe1—C9—C8	−165.1 (4)	C25—Fe2—C27—C28	−82.1 (4)
C8—C9—C10—C11	0.4 (7)	C29—Fe2—C27—C28	43.1 (8)
Fe1—C9—C10—C11	59.7 (5)	C31—Fe2—C27—C28	124.0 (4)
C8—C9—C10—Fe1	−59.3 (4)	C30—Fe2—C27—C28	80.7 (4)
C2—Fe1—C10—C9	156.2 (6)	C26—Fe2—C27—C28	−119.3 (5)
C3—Fe1—C10—C9	−166.0 (4)	C24—Fe2—C27—C26	81.7 (4)
C7—Fe1—C10—C9	81.6 (4)	C33—Fe2—C27—C26	−36.7 (10)
C8—Fe1—C10—C9	38.0 (4)	C32—Fe2—C27—C26	−74.4 (4)
C6—Fe1—C10—C9	−43.6 (8)	C25—Fe2—C27—C26	37.3 (3)
C11—Fe1—C10—C9	119.4 (6)	C29—Fe2—C27—C26	162.5 (6)
C5—Fe1—C10—C9	−82.2 (4)	C31—Fe2—C27—C26	−116.7 (4)
C4—Fe1—C10—C9	−124.1 (4)	C28—Fe2—C27—C26	119.3 (5)
C2—Fe1—C10—C11	36.8 (9)	C30—Fe2—C27—C26	−159.9 (3)
C3—Fe1—C10—C11	74.6 (5)	C26—C27—C28—C24	−0.4 (7)
C7—Fe1—C10—C11	−37.8 (4)	Fe2—C27—C28—C24	58.6 (4)
C8—Fe1—C10—C11	−81.4 (4)	C26—C27—C28—Fe2	−59.0 (4)
C6—Fe1—C10—C11	−163.1 (6)	C25—C24—C28—C27	0.8 (6)
C9—Fe1—C10—C11	−119.4 (6)	C23—C24—C28—C27	−172.2 (5)
C5—Fe1—C10—C11	158.4 (4)	Fe2—C24—C28—C27	−59.6 (4)
C4—Fe1—C10—C11	116.5 (4)	C25—C24—C28—Fe2	60.3 (4)
C9—C10—C11—C7	−0.4 (7)	C23—C24—C28—Fe2	−112.6 (5)
Fe1—C10—C11—C7	59.5 (4)	C24—Fe2—C28—C27	119.9 (5)
C9—C10—C11—Fe1	−59.9 (5)	C33—Fe2—C28—C27	162.6 (6)
C8—C7—C11—C10	0.3 (7)	C32—Fe2—C28—C27	−42.1 (10)
Fe1—C7—C11—C10	−59.7 (5)	C25—Fe2—C28—C27	81.1 (4)
C8—C7—C11—Fe1	59.9 (4)	C29—Fe2—C28—C27	−161.6 (4)
C2—Fe1—C11—C10	−166.6 (4)	C31—Fe2—C28—C27	−77.3 (4)
C3—Fe1—C11—C10	−124.1 (4)	C30—Fe2—C28—C27	−118.9 (4)
C7—Fe1—C11—C10	118.9 (6)	C26—Fe2—C28—C27	37.5 (4)
C8—Fe1—C11—C10	81.2 (4)	C33—Fe2—C28—C24	42.8 (7)
C6—Fe1—C11—C10	158.4 (8)	C32—Fe2—C28—C24	−161.9 (8)
C9—Fe1—C11—C10	37.2 (4)	C25—Fe2—C28—C24	−38.7 (3)
C5—Fe1—C11—C10	−51.0 (9)	C29—Fe2—C28—C24	78.6 (4)
C4—Fe1—C11—C10	−82.7 (5)	C31—Fe2—C28—C24	162.8 (3)
C2—Fe1—C11—C7	74.5 (5)	C30—Fe2—C28—C24	121.2 (3)
C3—Fe1—C11—C7	117.0 (4)	C26—Fe2—C28—C24	−82.3 (3)
C8—Fe1—C11—C7	−37.7 (4)	C27—Fe2—C28—C24	−119.9 (5)
C6—Fe1—C11—C7	39.5 (11)	C24—Fe2—C29—C30	125.3 (4)
C10—Fe1—C11—C7	−118.9 (6)	C33—Fe2—C29—C30	−118.6 (5)
C9—Fe1—C11—C7	−81.6 (4)	C32—Fe2—C29—C30	−81.1 (4)
C5—Fe1—C11—C7	−169.9 (6)	C25—Fe2—C29—C30	166.8 (3)
C4—Fe1—C11—C7	158.4 (4)	C31—Fe2—C29—C30	−37.6 (4)
Er1—O3—C12—O4	4.5 (5)	C28—Fe2—C29—C30	83.4 (4)
Er1—O3—C12—C13	−174.8 (4)	C26—Fe2—C29—C30	−163.9 (6)
Er1—O4—C12—O3	−4.5 (5)	C27—Fe2—C29—C30	52.5 (8)
Er1—O4—C12—C13	174.9 (4)	C24—Fe2—C29—C33	−116.0 (4)
O5—Er1—C12—O3	−12.8 (3)	C32—Fe2—C29—C33	37.5 (4)
O1—Er1—C12—O3	89.8 (3)	C25—Fe2—C29—C33	−74.6 (4)
O8—Er1—C12—O3	−85.8 (3)	C31—Fe2—C29—C33	81.0 (4)
O2^i^—Er1—C12—O3	−151.0 (3)	C28—Fe2—C29—C33	−157.9 (4)
O7—Er1—C12—O3	81.5 (5)	C30—Fe2—C29—C33	118.6 (5)
O4—Er1—C12—O3	175.6 (5)	C26—Fe2—C29—C33	−45.3 (9)
O1^i^—Er1—C12—O3	156.4 (3)	C27—Fe2—C29—C33	171.1 (6)
C1^i^—Er1—C12—O3	−177.3 (3)	C33—C29—C30—C31	−0.1 (7)
O5—Er1—C12—O4	171.7 (3)	Fe2—C29—C30—C31	59.3 (4)
O1—Er1—C12—O4	−85.7 (3)	C33—C29—C30—Fe2	−59.4 (4)
O8—Er1—C12—O4	98.6 (3)	C24—Fe2—C30—C29	−72.6 (4)
O2^i^—Er1—C12—O4	33.5 (3)	C33—Fe2—C30—C29	38.2 (4)
O7—Er1—C12—O4	−94.0 (5)	C32—Fe2—C30—C29	81.9 (4)
O3—Er1—C12—O4	−175.6 (5)	C25—Fe2—C30—C29	−35.9 (9)
O1^i^—Er1—C12—O4	−19.2 (3)	C31—Fe2—C30—C29	119.2 (6)
C1^i^—Er1—C12—O4	7.1 (3)	C28—Fe2—C30—C29	−115.5 (4)
O5—Er1—C12—C13	79 (6)	C26—Fe2—C30—C29	166.1 (6)
O1—Er1—C12—C13	−178 (6)	C27—Fe2—C30—C29	−157.9 (4)
O8—Er1—C12—C13	6(6)	C24—Fe2—C30—C31	168.2 (4)
O2^i^—Er1—C12—C13	−59 (6)	C33—Fe2—C30—C31	−81.1 (4)
O7—Er1—C12—C13	173 (5)	C32—Fe2—C30—C31	−37.4 (4)
O3—Er1—C12—C13	92 (6)	C25—Fe2—C30—C31	−155.1 (7)
O4—Er1—C12—C13	−93 (6)	C29—Fe2—C30—C31	−119.2 (6)
O1^i^—Er1—C12—C13	−112 (6)	C28—Fe2—C30—C31	125.2 (4)
C1^i^—Er1—C12—C13	−85 (6)	C26—Fe2—C30—C31	46.8 (8)
O3—C12—C13—C14	−174.4 (5)	C27—Fe2—C30—C31	82.8 (4)
O4—C12—C13—C14	6.3 (9)	C29—C30—C31—C32	0.2 (7)
Er1—C12—C13—C14	96 (6)	Fe2—C30—C31—C32	59.5 (4)
O3—C12—C13—C17	1.3 (9)	C29—C30—C31—Fe2	−59.3 (4)
O4—C12—C13—C17	−178.0 (5)	C24—Fe2—C31—C32	−154.0 (7)
Er1—C12—C13—C17	−88 (6)	C33—Fe2—C31—C32	−37.8 (4)
O3—C12—C13—Fe3	−85.6 (6)	C25—Fe2—C31—C32	41.4 (8)
O4—C12—C13—Fe3	95.1 (6)	C29—Fe2—C31—C32	−81.9 (4)
Er1—C12—C13—Fe3	−175 (5)	C28—Fe2—C31—C32	165.3 (4)
C21—Fe3—C13—C14	−169.3 (8)	C30—Fe2—C31—C32	−119.3 (6)
C17—Fe3—C13—C14	−119.5 (6)	C26—Fe2—C31—C32	79.8 (4)
C22—Fe3—C13—C14	156.5 (5)	C27—Fe2—C31—C32	123.1 (4)
C18—Fe3—C13—C14	115.4 (5)	C24—Fe2—C31—C30	−34.7 (10)
C19—Fe3—C13—C14	76.0 (5)	C33—Fe2—C31—C30	81.5 (4)
C20—Fe3—C13—C14	44.2 (10)	C32—Fe2—C31—C30	119.3 (6)
C15—Fe3—C13—C14	−37.9 (5)	C25—Fe2—C31—C30	160.7 (6)
C16—Fe3—C13—C14	−81.9 (5)	C29—Fe2—C31—C30	37.4 (4)
C21—Fe3—C13—C17	−49.8 (9)	C28—Fe2—C31—C30	−75.4 (5)
C22—Fe3—C13—C17	−84.0 (6)	C26—Fe2—C31—C30	−160.9 (4)
C18—Fe3—C13—C17	−125.1 (5)	C27—Fe2—C31—C30	−117.6 (4)
C19—Fe3—C13—C17	−164.6 (5)	C30—C31—C32—C33	−0.1 (7)
C20—Fe3—C13—C17	163.7 (9)	Fe2—C31—C32—C33	59.6 (4)
C15—Fe3—C13—C17	81.6 (5)	C30—C31—C32—Fe2	−59.7 (4)
C16—Fe3—C13—C17	37.6 (5)	C24—Fe2—C32—C31	162.5 (5)
C14—Fe3—C13—C17	119.5 (6)	C33—Fe2—C32—C31	118.8 (6)
C21—Fe3—C13—C12	69.5 (10)	C25—Fe2—C32—C31	−161.7 (4)
C17—Fe3—C13—C12	119.3 (7)	C29—Fe2—C32—C31	81.0 (4)
C22—Fe3—C13—C12	35.2 (7)	C28—Fe2—C32—C31	−45.5 (10)
C18—Fe3—C13—C12	−5.8 (7)	C30—Fe2—C32—C31	37.6 (4)
C19—Fe3—C13—C12	−45.3 (7)	C26—Fe2—C32—C31	−119.1 (4)
C20—Fe3—C13—C12	−77.0 (11)	C27—Fe2—C32—C31	−78.1 (5)
C15—Fe3—C13—C12	−159.2 (6)	C24—Fe2—C32—C33	43.7 (7)
C16—Fe3—C13—C12	156.9 (7)	C25—Fe2—C32—C33	79.5 (4)
C14—Fe3—C13—C12	−121.3 (7)	C29—Fe2—C32—C33	−37.9 (4)
C17—C13—C14—C15	0.6 (7)	C31—Fe2—C32—C33	−118.8 (6)
C12—C13—C14—C15	176.9 (5)	C28—Fe2—C32—C33	−164.3 (8)
Fe3—C13—C14—C15	59.5 (4)	C30—Fe2—C32—C33	−81.3 (4)
C17—C13—C14—Fe3	−58.9 (5)	C26—Fe2—C32—C33	122.1 (4)
C12—C13—C14—Fe3	117.4 (6)	C27—Fe2—C32—C33	163.1 (4)
C21—Fe3—C14—C15	48.2 (12)	C31—C32—C33—C29	0.1 (7)
C17—Fe3—C14—C15	−80.5 (5)	Fe2—C32—C33—C29	60.0 (4)
C22—Fe3—C14—C15	−171.0 (8)	C31—C32—C33—Fe2	−59.9 (4)
C13—Fe3—C14—C15	−118.5 (6)	C30—C29—C33—C32	0.0 (7)
C18—Fe3—C14—C15	158.0 (6)	Fe2—C29—C33—C32	−60.0 (4)
C19—Fe3—C14—C15	117.5 (6)	C30—C29—C33—Fe2	60.1 (4)
C20—Fe3—C14—C15	76.8 (6)	C24—Fe2—C33—C32	−160.0 (3)
C16—Fe3—C14—C15	−37.4 (5)	C25—Fe2—C33—C32	−117.4 (4)
C21—Fe3—C14—C13	166.7 (9)	C29—Fe2—C33—C32	119.1 (6)
C17—Fe3—C14—C13	37.9 (4)	C31—Fe2—C33—C32	37.7 (4)
C22—Fe3—C14—C13	−52.6 (10)	C28—Fe2—C33—C32	169.4 (5)
C18—Fe3—C14—C13	−83.5 (5)	C30—Fe2—C33—C32	81.4 (4)
C19—Fe3—C14—C13	−124.0 (4)	C26—Fe2—C33—C32	−77.3 (4)
C20—Fe3—C14—C13	−164.7 (4)	C27—Fe2—C33—C32	−49.4 (10)
C15—Fe3—C14—C13	118.5 (6)	C24—Fe2—C33—C29	80.9 (4)
C16—Fe3—C14—C13	81.1 (4)	C32—Fe2—C33—C29	−119.1 (6)
C13—C14—C15—C16	0.1 (8)	C25—Fe2—C33—C29	123.5 (4)
Fe3—C14—C15—C16	59.2 (6)	C31—Fe2—C33—C29	−81.4 (4)
C13—C14—C15—Fe3	−59.1 (4)	C28—Fe2—C33—C29	50.3 (8)
C21—Fe3—C15—C14	−163.0 (5)	C30—Fe2—C33—C29	−37.6 (4)
C17—Fe3—C15—C14	83.1 (5)	C26—Fe2—C33—C29	163.6 (4)
C22—Fe3—C15—C14	165.4 (13)	C27—Fe2—C33—C29	−168.5 (7)
C13—Fe3—C15—C14	38.4 (4)		

Symmetry codes: (i) −*x*+1, −*y*, −*z*+1.

### Hydrogen-bond geometry (Å, °)

**Table:**

Cg1 is the centroid of the [please define] ring.

**Table:**

*D*—H···*A*	*D*—H	H···*A*	*D*···*A*	*D*—H···*A*
C20—H20···Cg1^ii^	0.93	3.18	3.823 (8)	128.
O7—H7C···O4^i^	0.85	1.93	2.765 (5)	168.
O8—H8A···O6	0.82	1.79	2.574 (5)	160.
O9—H9···O6^iii^	0.82	1.87	2.682 (6)	172.
C7—H7···O4^i^	0.93	2.55	3.465 (7)	167.
C29—H29···O3	0.93	2.60	3.414 (7)	147.
C34—H34A···O2^i^	0.96	2.54	3.051 (9)	114.

Symmetry codes: (ii) −*x*+2, −*y*, −*z*; (i) −*x*+1, −*y*, −*z*+1;  (iii) −*x*+1, −*y*+1, −*z*+1.
